# Eyeless cave-dwelling *Leptonetela* spiders still rely on light

**DOI:** 10.1126/sciadv.adj0348

**Published:** 2023-12-20

**Authors:** Kai Wang, Jinhui Wang, Bing Liang, Jian Chang, Yang Zhu, Jian Chen, Ingi Agnarsson, Daiqin Li, Yu Peng, Jie Liu

**Affiliations:** ^1^ The State Key Laboratory of Biocatalysis and Enzyme Engineering of China, School of Life Sciences, Hubei University, Wuhan, Hubei 430062, China.; ^2^ Hubei Key Laboratory of Regional Development and Environmental Response, Faculty of Resources and Environmental Sciences, Hubei University, Wuhan, Hubei 430062, China.; ^3^ Faculty of Life and Environmental Sciences, University of Iceland, Sturlugata 7, 102 Reykjavik, Iceland.; ^4^ Department of Biological Sciences, National University of Singapore, Singapore 117543, Singapore.; ^5^ School of Nuclear Technology and Chemistry and Biology, Hubei University of Science and Technology, Xianning, Hubei 437100, China.

## Abstract

Subterranean animals living in perpetual darkness may maintain photoresponse. However, the evolutionary processes behind the conflict between eye loss and maintenance of the photoresponse remain largely unknown. We used *Leptonetela* spiders to investigate the driving forces behind the maintenance of the photoresponse in cave-dwelling spiders. Our behavioral experiments showed that all eyeless/reduced-eyed cave-dwelling species retained photophobic response and that they had substantially decreased survival at cave entrances due to weak drought resistance. The transcriptomic analysis demonstrated that nearly all phototransduction pathway genes were present and that all tested phototransduction pathway genes were subjected to strong functional constraints in cave-dwelling species. Our results suggest that cave-dwelling eyeless spiders still use light and that light detection likely plays a role in avoiding the cave entrance habitat. This study confirms that some eyeless subterranean animals have retained their photosensitivity due to natural selection and provides a case of mismatch between phenotype and genotype or physiological function in a long-term evolutionary process.

## Teaser

Behavioral and molecular evidences reveal that eyeless cave-dwelling *Leptonetela *spiders could detect light and still need light.

## INTRODUCTION

Subterranean animals living in perpetual darkness typically exhibit various phenotypic modifications, including eye degeneration and disappearance of skin pigments (*1*, *2*). The evolution of eye loss has fascinated biologists since Darwin commented on it in *On the Origin of Species* (*3*). Previous studies have suggested that eye loss in subterranean animals may be the result of neutral or adaptive evolution (*4*). Specifically, selection may drive eye loss in cave animals because functional eyes require energy allocation that can be costly in low nutrition environment (*5*). In either case, animals in constant darkness would be expected to lose their photoresponsive ability (*6*, *7*). However, a growing number of eye-degraded or eyeless obligatory subterranean animals have been demonstrated to maintain photoresponse, including nematodes (*8*), amphipods (*9*–*12*), beetles (*13*, *14*), isopods (*15*, *16*), and cavefishes (*17*, *18*). The conflict between eye loss and maintenance of photoresponse has therefore attracted attention.

Why would photoreceptor activity be maintained in eyeless animals living in complete darkness? There are two mutually exclusive hypotheses regarding the evolutionary processes involved in visual maintenance in lightless environments. Photoresponse behavior may be maintained by natural selection via selective benefits. These may include habitat choice, avoidance of predation or competition from surface dwellers, or benefits of light avoidance (*19*), as demonstrated by eyeless cave-dwelling amphipods avoiding wandering out of the cave environment (*20*). Alternatively, the neutral hypothesis posits that the loss or maintenance of traits results from the accumulation of random mutations in genes that are no longer under purifying selection (*21*). For example, studies of photophobic behavior in six species of eyeless aquatic cave beetles showed that the photophobic response was absent in five species, while one species retained this trait apparently by random chance (*14*). However, previous studies have rarely provided evidence to support the hypothesis that photoresponse behavior is retained because of natural selection or neutral evolution in lightless environments for subterranean animals, and these hypotheses have never been tested in any group using modern molecular evolutionary tools.

**Fig. 1.**
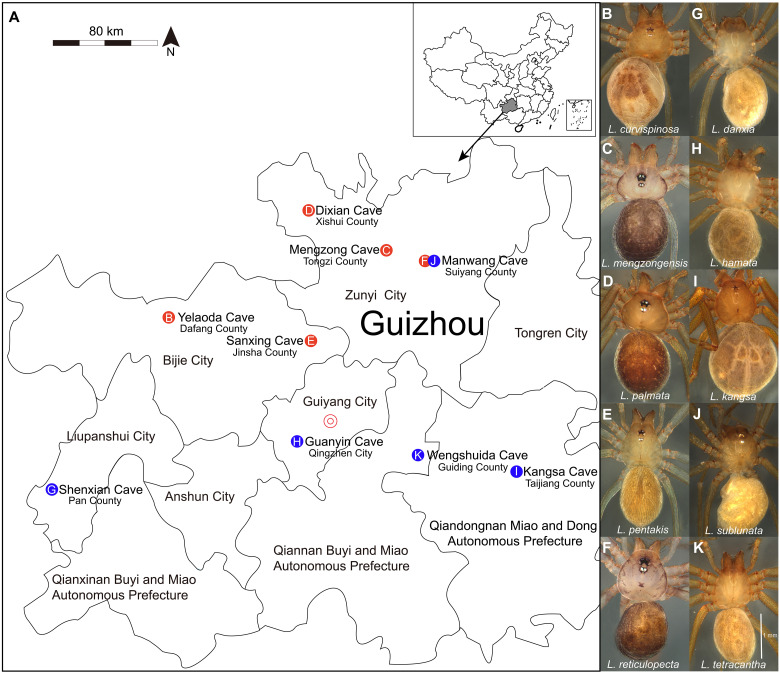
Collection sites and phenotypes of entrance and cave-dwelling *Leptonetela* species. (**A**) A map showing the distribution of caves in Guizhou Province, China; red and blue circles separately indicate the collection locations of entrance and cave-dwelling *Leptonetela* species. (**B** to **F**) Phenotypes of five entrance *Leptonetela* species (*L. curvispinosa*, *L. mengzongensis*, *L. palmata*, *L. pentakis*, and *L. reticulopecta*). The images display the recognition of six intact eyes with completely eye pigment in five entrance species. (**G** to **K**) Phenotypes of five cave-dwelling *Leptonetela* species (*L. danxia*, *L. hamata*, *L. kangsa*, *L. tetracantha*, and *L. sublunata*). The images show highly reduced eyes with only six white spots in two cave-dwelling species [(I) and (J)] and the completely absence of peripherally detectable eyes in three cave-dwelling species [(G), (H), and (K)]. The scale in (K) could be applied to all images.

Spiders are an ancient group that originated approximately 400 million years (Ma) ago (*22*), and modern spiders show a variety of functional adaptations to cave environments. Spiders represent the most species-rich lineage of top predators in cave ecosystems, with nearly 1000 recorded species belonging to over 48 families (*23*). Accordingly, subterranean spiders have served as models for physiological, ecological, behavioral, evolutionary, and biogeographic studies. However, cave adaptations in spiders such as eye loss remain poorly understood, and our knowledge of the process largely relies on morphological reports. Spider visual systems have received increasing attention in recent years due to their diversity. With the increasing availability of transcriptomic data, several recent studies have explored the molecular and genetic bases of spider vision (*24*–*27*). *Leptonetela* (Araneae: Leptonetidae) spiders are broadly distributed in the karst landforms of southwest China and the Balkan Peninsula in Europe. Unlike most spiders that have eight eyes (*24*), *Leptonetela* species have only six eyes due to the secondary loss of the anterior median eyes (*28*). Because the anterior median eyes of spiders are generally involved in identifying and stalking prey (*24*), their losses indicate that visual-dependent prey pursuit may have been generally regressed in *Leptonetela* species. As they spend their entire life cycles in cave environment, *Leptonetela* spiders generally exhibit various degrees of eye reduction, from six intact eyes to highly reduced/reduced-eyed forms (having only six white spots) or eyeless (no organized eye structures), which were categorized as macrophthalmic species, microphthalmic species, and anophthalmic species in a previous study (*29*). For convenience, *Leptonetela* species frequently distributed in twilight entrances will be called “entrance species” (Fig. 1, B to F) and that distributed in the dark zone will be referred to as “cave-dwelling species” (Fig. 1, G to K) hereafter in this study. Therefore, *Leptonetela* spiders are ideal models for investigating the photoresponsive ability and the factors that may drive evolutionary change in such an ability.

We conducted a comparative study to investigate photoresponse behavior and assessed the relative roles of natural selection and neutral evolution in the maintenance of phototransduction systems in cave-dwelling *Leptonetela* spiders by integrating field behavior assays, transcriptomic data, and molecular evolutionary analyses. We tested the two abovementioned hypotheses regarding the maintenance of photoresponse behavior in eyeless subterranean animals. We first conducted behavioral tests in local caves to examine whether cave-dwelling *Leptonetela* spiders had a notable photoresponse. Second, we performed field and laboratory experiments to test whether survival rates of cave-dwelling *Leptonetela* species significantly decreased outside the cave environment to determine whether the maintenance of photoresponse potentially enhances the fitness of cave-dwelling spiders. Third, we annotated 13 core phototransduction pathway genes (PPGs) to verify their presence in all *Leptonetela* spiders. Last, we used a molecular evolutionary analysis to test whether these PPGs of cave-dwelling spiders have been under natural or neutral selection.

## RESULTS

### Cave-dwelling *Leptonetela* species showed negative phototactic responses

To test whether eyeless or eye-degraded *Leptonetela* spiders could interact with light, we conducted behavioral experiments in the field using five cave-dwelling species and five entrance species (table S1). The results showed that the majority of individuals (85 to 100%) of each of the 10 tested species chose to stay in dark areas of the boxes when placed at the cave entrance regardless of the degree of eye degradation (*P* < 0.05; Table 1). Individuals (35 to 60%) did not show a preference for dark areas after being transferred into the deep cave (*P* > 0.05; Table 1), indicating that *Leptonetela* spiders randomly chose areas of the boxes in the lightless environment. However, for each *Leptonetela* species, significantly more individuals chose to stay in dark areas when exposed to the twilight of the cave entrance than those placed in the deep cave (*P* < 0.05; Table 1). The results of the behavioral experiments demonstrated that both entrance and cave-dwelling *Leptonetela* spiders exhibited negative phototactic behavior, revealing the ability to detect light in eyeless and eye-degraded spiders.

**Table 1.** Results of behavioral experiments of photoresponse for five entrance and five cave-dwelling species. *B*, Number of individuals that stayed in the bright areas of boxes; *D*, number of individuals that stayed in the dark areas of boxes; I, tested in the deep cave area of Manwang Cave; O, tested at the entrance of Manwang Cave; *R*, percentages of individuals that stayed in the dark areas of boxes; RD, tested individuals were assumed to on average stay in the dark areas and bright areas of boxes. *P* values lower than 0.05 are shown in bold.

Species	Treatments	*D*	*B*	*R*	Comparisons	χ^2^	*P* values
*L. curvispinosa*	O	19	1	95%	O versus RD	10.16	**1.44 × 10** ^**−3**^
	I	12	8	60%	I versus RD	0.40	0.53
	O versus I					7.03	**8.04 × 10^−3^**
*L. danxia*	O	17	3	85%	O versus RD	5.58	**1.80 × 10** ^**−2**^
	I	8	12	40%	I versus RD	0.40	0.53
	O versus I					8.64	**3.00 × 10** ^**−3**^
*L. mengzongensis*	O	20	0	100%	O versus RD	13.33	**2.61 × 10** ^**−4**^
	I	8	12	40%	I versus RD	0.40	0.53
	O versus I					17.14	**3.50 × 10^−5^**
*L. hamata*	O	19	1	95%	O versus RD	10.16	**1.44 × 10^−3^**
	I	7	13	35%	I versus RD	0.92	0.34
	O versus I					15.82	**7.00 × 10** ^**−5**^
*L. palmata*	O	17	3	85%	O versus RD	5.58	**1.80 × 10** ^**−2**^
	I	10	10	50%	I versus RD	0	1
	O versus I					5.58	**1.80 × 10** ^**−2**^
*L. kangsa*	O	18	2	90%	O versus RD	7.62	**5.76 × 10** ^**−3**^
	I	9	11	45%	I versus RD	0.10	0.75
	O versus I					9.23	**2.00 × 10** ^**−3**^
*L. pentakis*	O	19	1	95%	O versus RD	10.16	**1.44 × 10** ^**−3**^
	I	11	9	55%	I versus RD	0.10	0.75
	O versus I					8.53	**3.49 × 10** ^**−3**^
*L. sublunata*	O	19	1	95%	O versus RD	10.16	**1.44 × 10** ^**−3**^
	I	10	10	50%	I versus RD	0.00	1
	O versus I					10.16	**1.44 × 10** ^**−3**^
*L. reticulopecta*	O	20	0	100%	O versus RD	13.33	**2.61 × 10** ^**−4**^
	I	9	11	45%	I versus RD	0.10	0.75
	O versus I					15.17	**9.80 × 10^−5^**
*L. tetracantha*	O	19	1	95%	O versus RD	10.16	**1.44 × 10^−3^**
	I	7	13	35%	I versus RD	0.92	0.34
	O versus I					15.17	**9.80 × 10** ^**−5**^

### Cave-dwelling *Leptonetela* species had lower survival at the dry cave entrance due to weak drought resistance

To test whether maintenance of negative phototaxis that allows the spiders to avoid the dry cave entrance environment can have positive fitness consequences and thus may reflect natural selection in eyeless or eye-degraded cave-dwelling *Leptonetela* spiders, we conducted field experiments to compare survival rates between spiders in their native deep cave and cave entrance using two cave-dwelling species (*L. danxia* and *L. sublunata*) and two entrance spider relatives (*L. palmata* and *L. reticulopecta*). For cave-dwelling species, our results showed that all individuals (*L. sublunata*, *n* = 20; *L. danxia*, *n* = 15) survived (100%) in the deep cave, but only 17 of 32 individuals (*L. sublunata*, 53.13%) and 2 of 15 individuals (*L. danxia*, 13.33%) survived at the cave entrance (Table 2). The survival rate of cave-dwelling species was significantly lower at the cave entrance than that in the deep cave (*P* < 0.001; Table 2). However, all individuals of two entrance species (*L. palmata* and *L. reticulopecta*) survived (100%) both at the cave entrance and in the deep cave (χ^2^ = 0, *P* = 1) (Table 2). These results indicate that it may be lethal to leave the deep cave even for a short time for the cave-dwelling spiders.

**Table 2.** Survival rates of two entrance species and two cave-dwelling species. *D*, Number of individual deaths; I, dark zone of a deep cave; O, twilight zone of a cave entrance; *R*, survival rate; *S*, number of individuals that eventually survived. *P* values lower than 0.05 are shown in bold.

Treatments	Species	Habitats	*S*	*D*	*R*	χ^2^	*P* values
Without water supply	*L. danxia*	I	15	0	100%	22.94	**2.00 × 10^−6^**
		O	2	13	13.33%		
	*L. sublunata*	I	20	0	100%	13.18	**2.84 × 10^−4^**
		O	17	15	53.13%		
Without water supply	*L. palmata*	I	15	0	100%	0	1
		O	15	0	100%		
	*L. reticulopecta*	I	10	0	100%	0	1
		O	12	0	100%		
With water supply	*L. danxia*	I	15	0	100%	0	1
		O	15	0	100%		
	*L. sublunata*	I	10	0	100%	0	1
		O	12	0	100%		
With water supply	*L. palmata*	I	15	0	100%	0	1
		O	15	0	100%		
	*L. reticulopecta*	I	20	0	100%	0	1
		O	30	0	100%		

On the basis of our field experience, cave-dwelling spiders are vulnerable to arid environments. The relative humidity (RH) in deep caves often surpasses 99%, while the RH near the cave entrance is usually below 90%. We hypothesized that lower survival rates of cave-dwelling *Leptonetela* spiders at the cave entrance were largely due to the relatively low humidity. To test it, we thus conducted additional field experiments to examine the spiders’ drought resistance using two cave-dwelling species (*L. danxia* and *L. sublunata*) and two entrance spider relatives (*L. palmata* and *L. reticulopecta*). When water was supplied, all tested individuals of all four species survived (100%) regardless of the habitat (χ^2^ = 0, *P* = 1) (Table 2). Providing water therefore reversed the poor survival of cave-dwelling spiders in the cave entrance environment (movie S1).

**Fig. 2.**
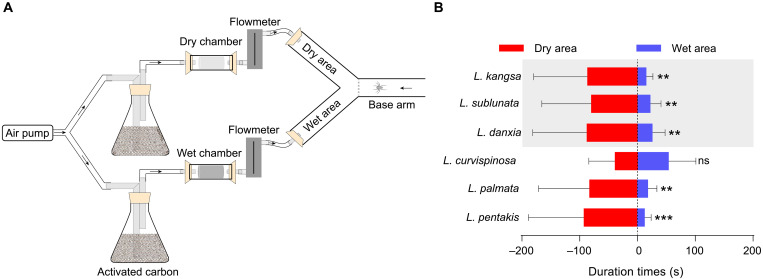
Relative humidity (RH) choice experiments for *Leptonetela* species in the laboratory. (**A**) A diagram showing the RH choice experiments using the Y-tube in the laboratory. (**B**) The results of RH choice test for *Leptonetela* species in the laboratory. The significant differences of duration times between two areas (dry and wet areas) were compared using a Student’s *t* test (paired, two-tailed; ^ns^*P* > 0.05, **P < 0.01, and ***P < 0.001). Cave-dwelling species are shown on a gray background. Data are represented as the means ± SEM from 20 spider individuals for each *Leptonetela* species.

To test whether *Leptonetela* species are able to detect humidity and prefer a higher humid environment, we conducted the RH choice experiment using a Y-tube with two different RH levels (Fig. 2A and movie S2). We tested it with three cave-dwelling species (*L. danxia*, *L. kangsa*, and *L. sublunata*) and found that the majority of individuals stayed in the dry areas significantly longer than they did in the wet areas (*L. danxia*, paired *t* tests: *t* = 3.124, *P* < 0.01; *L. kangsa*, *t* = 3.869, *P* < 0.01; *L. sublunata*, *t* = 3.529, *P* < 0.01) (Fig. 2B). We also tested it with three entrance species (*L. curvispinosa*, *L. palmata*, and *L. pentakis*) and found that individuals of two entrance species stayed in the dry areas significantly longer than in the wet areas (*L. palmata*, *t* = 3.699, *P* < 0.01; *L. pentakis*, *t* = 6.303, *P* < 0.001). However, individuals of *L. curvispinosa* stayed in the dry areas as long as they stayed in the wet areas (*t* = 0.347, *P* = 0.733) (Fig. 2B). Our results indicated that all cave-dwelling and some entrance species were able to sense humidity and showed a preference for dry environments.

### Phylogenetic reconstruction of *Leptonetela* species based on transcriptomic data

We obtained 10 de novo assembled transcriptomes for five entrance species (*L. curvispinosa*, *L. mengzongensis*, *L. palmata*, *L. pentakis*, and *L. reticulopecta*) and five cave-dwelling species (*L. danxia*, *L. hamata*, *L. kangsa*, *L. sublunata*, and *L. tetracantha*) (table S2). The completeness of 10 de novo assembly transcriptomes was assessed by BUSCO (Benchmarking Universal Single-Copy Orthologs) (*30*), and 76.3 to 94.8% were complete sequences of the 1013 genes used for benchmarking in Arthropoda. Between 2.4 and 10.3% of the sequences were fragmented, and 2.8 to 13.4% were missing (table S3). The aligned sequences amounted to 1,269,328 base pairs (bp) based on 1436 single-copy orthologs from 11 individuals belonging to 10 *Leptonetela* species and *Calileptoneta californica* (Leptonetidae) that was chosen as the outgroup as a member of the genus closest to *Leptonetela* with publicly available transcriptomic data (*31*).

**Fig. 3.**
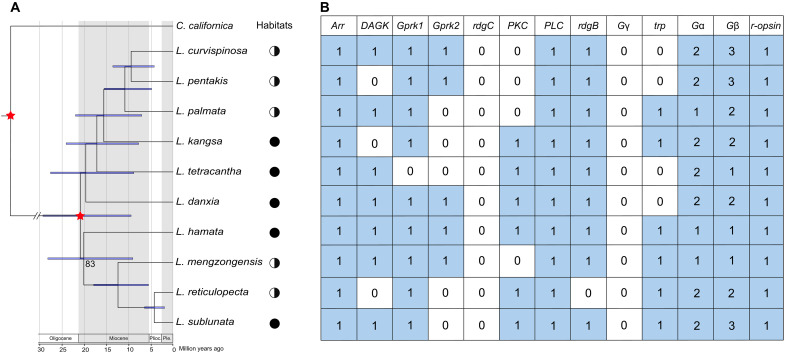
Phylogenetic tree of *Leptonetela* spiders and phototransduction pathway genes (PPGs) identified in 10 *Leptonetela* species. (**A**) The phylogenetic tree of *Leptonetela* species. The red stars represent two time-calibration points used in the analysis. The bootstrap support values of 100% were not shown in the phylogenetic tree. *C. californica* (Leptonetidae) was chosen as outgroup. Half-black circles represent the twilight entrance of the inhabited cave; black circles represent the dark zone of the deep cave. (**B**) PPGs identified in 10 *Leptonetela* species. Blue and white marks represent the respective orthologs of PPGs that are present and absent in transcriptomes of the 10 *Leptonetela* spider individuals. The numbers represent the number of orthologs of PPGs.

The topology of our phylogenetic tree was very stable, and almost all nodes were well supported (bootstrap value = 100%) (Fig. 3A). Thus, the phylogeny served as a strong tool to conduct a selective pressure analysis of PPGs of *Leptonetela* species. Our dated phylogeny showed that the common ancestor of targeted *Leptonetela* spiders was estimated to have existed during the Miocene, before 20.8 Ma ago [95% highest posterior density (HPD): 9.4 to 29.2 Ma ago], consistent with the Chinese *Leptonetela* ancestor originating about 19.0 Ma ago according to the world *Leptonetela* phylogeny (*31*). These 10 *Leptonetela* species were divided into two major clades: one containing two entrance species (*L. mengzongensis* and *L. reticulopecta*) and two cave-dwelling species (*L. hamata* and *L. sublunata*) and the other consisting of three entrance species (*L. curvispinosa*, *L. palmata*, and *L. pentakis*) and three cave-dwelling species (*L. danxia*, *L. kangsa*, and *L. tetracantha*) (Fig. 3A). No cave-dwelling *Leptonetela* species were monophyletic for either clade, indicating that the habitat shift between the cave entrances and the deep caves may have occurred independently multiple times. This provides a natural evolutionary experiment and increases the power of our test of the consequences of this habitat shift for eye loss and maintenance of photosensitivity. We cannot specify the original habitat of *Leptonetela* spiders due to our limited sampling of less than 10% of known species of *Leptonetela*. However, the southern China karst caves have been thought of as suitable refugia for cave-dwelling spiders during periods of unfavorable climatic conditions (*32*). Our dated phylogeny indicated that surface-adapted *Leptonetela* ancestors frequently moved into caves during the mid-Miocene (about 20.0 to 15.5 Ma ago) and in the early Pliocene (about 4.2 Ma ago) (Fig. 3A), consistent with the habitat shifts (from surface to cave) of cave-dwelling *Nesticella* spiders (*33*).

### PPGs are generally present in cave-dwelling *Leptonetela* species

A total of 13 core genes belonging to the phototransduction pathway were annotated on the basis of our transcriptomic data for each *Leptonetela* species according to the definition in the phylogenetically informed annotation (PIA) tool (*34*). Our results revealed that a nearly complete phototransduction pathway was present, not only in entrance spiders but also in eyeless cave-dwelling spiders (Fig. 3B and table S4). With the exceptions of the *G protein* γ*-subunit* (*G*γ) and *Rhodopsin phosphatase retinal degeneration C* (*rdgC*), which were not found in any *Leptonetela* spiders, other PPGs were found in both entrance and cave-dwelling species. In addition, we found that five PPGs, *Arrestin* (*Arr*), *Rhodopsin* (*r-opsin*), *Phospholipase C* (*PLC*), *G protein* α-*subunit* (Gα), and *G protein* β*-subunit* (*G*β), were present in all 10 *Leptonetela* species. The remaining PPGs, *Diacylglycerol kinase* (*DAGK*), *G protein–coupled receptor kinase 1* (*Gprk1*), *Gprk2*, *Protein C kinase* (*PKC*), *Retinal degeneration B* (*rdgB*), and *Transient receptor potential protein* (*trp*), were annotated in at least one species of both entrance and cave-dwelling spiders. Results of the PPGs annotation further supported the hypothesis that cave-dwelling spiders may have retained the ability to detect light although their eyes have been highly reduced or completely lost.

### PPGs of *Leptonetela* species are under purifying selection

Six PPGs present in at least three entrance and three cave-dwelling *Leptonetela* species were chosen to test whether they were under natural selection in cave-dwelling spiders. First, under the assumption of all branches having a uniform ω (model A in Table 3), ω for all six PPGs was estimated to be significantly less than 1 (*P* < 0.001; see the comparison of model B versus model A in Table 3). This result suggests that all tested PPGs were under strong purifying selection in these species. Second, the model allowing different ω values between entrance and cave-dwelling species (model C in Table 3) did not fit the data significantly better than the model that assumed that all branches had a uniform ω (model A in Table 3) for all the PPGs (*P* > 0.05; see the comparison between model A versus model C in Table 3), suggesting that PPGs are generally under similar levels of purifying selection between entrance and cave-dwelling species. Third, we found that model C yielded a significantly better fit than model D, which assumed that the ω values for cave-dwelling species were equal to 1 for all tested PPGs, suggesting that the ω values of the six PPGs for cave-dwelling species were significantly less than 1 (*P* < 0.001; see the comparison of model D versus model C in Table 3). Fourth, we found that model E (Table 3), in which each branch had its own value of ω, was not significantly better than model A, which assumed that all branches had a uniform ω (*P* > 0.05; see the comparison of model A versus model E in Table 3), suggesting that the variation in ω among branches of *Leptonetela* species was minimal. Furthermore, we ran the RELAX program by setting cave-dwelling species as test branches and entrance species as reference branches (*35*). Our results do not imply relaxed selection for almost all tested PPGs of cave-dwelling species compared with their entrance relatives after contrasting the alternative model with the null model (*P* > 0.05; table S5) except for the *Arr* gene. Additional analyses based on the partitioned exploratory model found no signals of relaxed selection in the six tested PPGs (*P* > 0.05; table S5).

**Table 3.** Likelihood ratio tests of selective pressures on phototransduction pathway genes (PPGs)1 in *Leptonetela* species. (A) All branches have a uniform ω_0_ ≠ 1. (B) All branches have a fixed ω_0_ = 1. (C) Cave-dwelling branches have ω_1_, and entrance branches have ω_2_. (D) Cave-dwelling branches have a fixed ω_0_ = 1; entrance branches have their own ω. (E) Each branch has its own ω.

Genes	Models	ω (*d*_N_/*d*_S_)	ln L*	np^†^	Models compared	2Δ(ln*L*)‡	*P* values§
*G*α	A	ω_0_ = 0.008	−1311.89	18	B versus A	151.44	**8.41 × 10^−35^**
	B	ω_0_ = 1	−1387.61	17			
	C	ω_1_ = 0.0272, ω_2_ = 0.0001	−1309.23	20	A versus C	5.31	0.070
	D	ω_1_ = 1, ω_2_ = 0.0001	−1332.09	18	D versus C	45.71	**1.19 × 10^−10^**
	E	Variable ω by branch	−1301.77	33	A versus E	20.23	0.163
*PLC*	A	ω_0_ = 0.0366	−6932.84	20	B versus A	429.21	**3.36 × 10^−184^**
	B	ω_0_ = 1	−7351.71	19			
	C	ω_1_ = 0.0466, ω_2_ = 0.0293	−6931.89	22	A versus C	0.88	0.386
	D	ω_1_ = 1, ω_2_ = 0.0296	−7119.93	20	D versus C	376.08	**2.17 × 10^−82^**
	E	Variable ω by branch	−6923.35	37	A versus E	9.23	0.330
*r-opsin*	A	ω_0_ = 0.0276	−2816.89	20	B versus A	398.21	**2.12 × 10^−142^**
	B	ω_0_ = 1	−3139.64	19			
	C	ω_1_ = 0.0393, ω_2_ = 0.0176	−2814.38	22	A versus C	0.66	0.082
	D	ω_1_ = 1, ω_2_ = 0.0113	−2934.60	20	D versus C	240.43	**6.19 × 10^−53^**
	E	Variable ω by branch	−2806.98	37	A versus E	10.19	0.283
*rdgB*	A	ω_0_ = 0.03	−1614.01	14	B versus A	328.08	**3.68 × 10^−61^**
	B	ω_0_ = 1	−1750.13	13			
	C	ω_1_ = 0.0418, ω_2_ = 0.0169	−1613.15	16	A versus C	0.04	0.424
	D	ω_1_ = 1, ω_2_ = 0.013	−1672.44	14	D versus C	118.59	**1.78 × 10^−26^**
	E	Variable ω by branch	−1606.83	25	A versus E	3.73	0.214
*G*β	A	ω_0_ = 0.0869	−830.58	16	B versus A	53.04	**9.08 × 10^−22^**
	B	ω_0_ = 1	−876.53	15			
	C	ω_1_ = 0.0697, ω_2_ = 0.1114	−830.20	18	A versus C	0.00	0.688
	D	ω_1_ = 1, ω_2_ = 0.1159	−859.93	16	D versus C	118.59	**1.78 × 10^−26^**
	E	Variable ω by branch	−820.45	29	A versus E	0.00	0.089
*Arr*	A	ω_0_ = 0.024	−2726.79	18	B versus A	393.59	**8.81 × 10^−141^**
	B	ω_0_ = 1	−3045.82	17			
	C	ω_1_ = 0.0331, ω_2_ = 0.0184	−2725.62	20	A versus C	23.43	0.311
	D	ω_1_ = 1, ω_2_ = 0.0134	−2825.25	18	D versus C	199.26	**5.39 × 10** ^**−44**^
	E	Variable ω by branch	−2716.95	33	A versus E	20.70	0.185

*The natural logarithm of the likelihood value.

†Number of parameters.

‡Twice the difference in ln *L* between the two models compared.

§*P* values lower than 0.05 are shown in bold.

## DISCUSSION

In this study, we conducted behavioral experiments and measured survival rates in local caves to minimize the impacts of factors other than light. Although energy-costly eyes were highly reduced or lost in cave-dwelling *Leptonetela* spiders, which spend their entire life cycles in the complete absence of light, our results demonstrated that they could detect light, and light cues may be used to avoid the perilously dry environment outside the cave. The annotation of core PPGs based on transcriptomic data suggests that cave-dwelling *Leptonetela* spiders have retained a nearly complete set of PPGs as in the entrance spiders. The molecular evolutionary analysis showed strong purifying selection on PPGs of cave-dwelling *Leptonetela* spiders. Therefore, our study provides evidence supporting the hypothesis that the phototransduction system of cave-dwelling eyeless *Leptonetela* spiders may have been under purifying selection rather than being a phylogenetic relic. Our results thus refute the neutral hypothesis.

*Leptonetela* spiders are small cryptozoic spiders that build sheet webs for capturing prey in twilight or lightless environment, such as leaf litter, rotting logs, rock crevices, and caves (*31*). Light is suggested to be the primary selective force driving the evolution of eyes of cave animals, thus, eyes are often reduced or lost as cave preadaptation in many litter-dwelling arthropods (*36*–*38*). *Leptonetela* spiders have lost anterior median eyes that are generally involved in identifying and stalking prey in spiders, likely due to their twilight or lightless habitats. In addition, cave-dwelling *Leptonetela* spiders living in lightless deep caves exhibit various degrees of eye reduction (highly reduced or eyeless) compared to their entrance spider relatives that have six intact eyes. Thus, *Leptonetela* spiders provided an ideal system for studying the evolution of eyes and visual systems.

This study provides evidence demonstrating negative phototaxis in cave-dwelling spiders, a highly diverse group that plays a critical role in cave ecosystems as top predators (*23*). Negative phototaxis has frequently been found in other subterranean animals. For example, the cave-dwelling carrion beetle *Ptomaphagus hirtus* that has highly reduced eyes nonetheless displays strongly negative phototaxis and maintains a reduced but functional phototransduction system, as shown by transcriptomic data (*13*). However, Langille *et al.* (*14*) reported that five of six subterranean water beetles completely lacked phototactic responses, and the authors proposed negative phototaxis as a preadaptation to living in permanent darkness for ancestral cave-dwelling animals. We speculate that drought resistance may play an important role in the retention of PPGs in *Leptonetela* spiders. Our results from the survival experiments indicate that cave-dwelling spiders have poor survival when moving outside the cave without a water supply, and we hypothesized that this was due to the dry environment. This hypothesis was tested and supported by the data obtained from our field experiments, wherein survival rates of the vulnerable cave-dwelling *Leptonetela* spiders were markedly enhanced outside the cave when given a water supply. Light may be a key cue for avoiding a dry habitat for terrestrial cave-dwelling arthropods such as spiders and carrion beetles. This may also explain why eyeless amphipods (*9*) and soil animals (*39*, *40*) remain sensitive to light. However, it is unnecessary for water beetles (*14*) to detect light to find humid environments. Therefore, we conclude that the evolution of visual systems in terrestrial cave-dwelling animals is divergent across taxa and may be partially related to drought resistance. We therefore hypothesize that drought resistance plays an important role in the retention of light perception in cave-dwelling *Leptonetela* spiders. While our experimental setup does not allow us to rule out other reasons such as avoidance of predation and competition with entrance spiders (*9*), the *Leptonetela* spiders clearly both avoid light and suffer in the dry cave entrance environments.

Our results obtained from the RH choice experiments suggest that both entrance and cave-dwelling *Leptonetela* spiders are able to sense humidity. All three tested cave-dwelling *Leptonetela* species exhibited an apparent preference for lower RH (Fig. 2B). In the deep caves, atmosphere is always water-saturated, and rock surfaces are often covered with a film of water. Dripping water and pools of water are also common in the deep caves (*41*). However, *Leptonetela* spiders are so small (1 to 3 mm) that even one dripping water could be deadly to them (*42*). Probably, a preference of relatively lower humidity may play a crucial role in keeping away from mortally liquid water for cave-dwelling *Leptonetela* spiders and some entrance spiders living in high RH environments. Regardless, our results negate the hypothesis that sensors for humidity/water may assist cave-dwelling *Leptonetela* spiders in avoiding the fatal dry cave entrance environments. Furthermore, because temperature is seasonally changed outside caves and in caves (*43*), it is persuasive that temperature is not a stable clue for the cave-dwelling spiders to choose deep caves as suitable habitats. Thus, we suggest that light intensity is probably the main clue for the cave-dwelling spiders to distinguish between deep cave and cave entrance environments and that avoidance of light may prevent cave-dwelling spiders from wandering into cave entrances, with negative survival consequences.

One may argue that photoresponse is vestigial for *Leptonetela* spiders and the absence of apparent mutations in PPGs is due to not enough time for neutral mutations to accumulate within them. In a previous study, researchers tested photophobic behavior in six species of eyeless aquatic cave beetles that have been isolated in lightless cave for more than 3 Ma, and they found that photoresponse was absent in five of the six tested species, while the other species retained this trait apparently by random chance (*14*). The study suggests that photoresponse is readily lost in eyeless subterranean animals if natural selection is not at play in maintaining it. However, our results have shown that all five tested cave-dwelling *Leptonetela* species with at least 4.2 Ma of independent cave adaptation exhibited negative phototactic behavior (Table 1). We can conclude that photoresponse may not be vestigial maintenance in cave-dwelling *Leptonetela* species.

Fisher argued that increases in mean fitness of populations resulted from natural selection (*44*). Although this notion has long been debated in population genetics, it has generally been embraced in other fields of biology (*45*). Natural selection normally increases the mean fitness of populations (*46*). Fitness generally involves the ability of organisms to survive and reproduce in their habitats (*47*). We found that the survival rate of cave-dwelling *Leptonetela* spiders significantly decreased when they were transplanted from the deep cave to the cave entrance, suggesting an important role of the ability to detect light in survival and thus being associated with fitness. Therefore, we provide evidence that negative phototaxis of cave-dwelling *Leptonetela* spiders is essential for their survival and is maintained through natural selection.

**Fig. 4.**
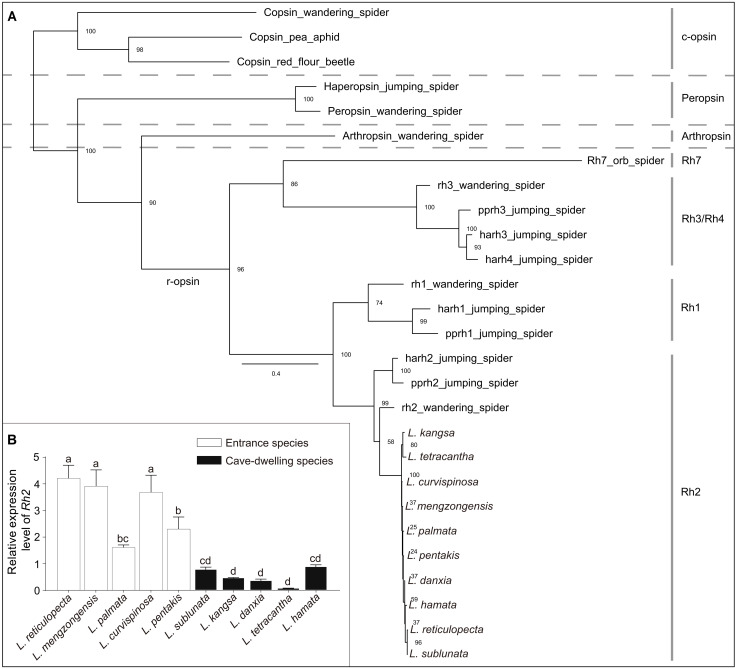
Phylogenetic tree of opsins and quantitative reverse transcription PCR of the *Rh2* gene in *Leptonetela* species. (**A**) Phylogenetic tree of opsins in spiders. Numbers at nodes are the maximum-likelihood (ML) bootstrap values, shown as percentages. The four major clades of opsin (r-opsin, arthropsin, peropsin, and c-opsin) were marked using dashed lines. (**B**) The relative expression level of *Rh2* in 10 *Leptonetela* species. The significant differences of relative expression level were compared using analysis of variance (ANOVA), followed by Duncan multiple comparison tests. Different lower case letters denote significant differences between comparisons. Data are represented as the means ± SEM from three biological repetitions.

We only annotated blue-sensitive r-opsin Rh2 in all *Leptonetela* transcriptomes (Fig. 4A), and this gene was found to be under strong purifying selection in both entrance and cave-dwelling *Leptonetela* species (*48*). However, we did not find any other r-opsin in the transcriptomes of *Leptonetela* species (e.g., Rh1 sensitive to long-wavelength light or Rh3 and Rh4 sensitive to ultraviolet light) that were previously identified in other spider families (*48*–*50*). These findings suggest that it may be essential for both entrance and cave-dwelling species to detect blue light. Previous research has demonstrated that the blue light wavelength (450 nm) has the lowest attenuation coefficient in karst caves (*51*). Although entrance spiders live in the environments with light-dark cycles, they are usually found in rock crevices in the twilight zone of cave entrance where limited light can reach. Perhaps, that blue light may be more accessible to them, and perceiving blue light thus becomes important for entrance spiders. Alternatively, the ability to detect blue light being retained in cave-dwelling *Leptonetela* spiders may facilitate discriminating the boundary between light and dark environments in caves and thus be an adaptation for avoiding the fatal dry cave entrance environment. Furthermore, while *Rh2* gene was expressed in all five entrance and five cave-dwelling *Leptonetela* species, there was a notable difference in the relative expression level of the *Rh2* gene among species of *Leptonetela* (Fig. 4B). In general, the expression of the *Rh2* gene was markedly diminished in the eyeless/reduced-eyed cave-dwelling species compared to the entrance species (*t* = 6.430, *P* < 0.001). These findings align with prior research that has also demonstrated a significant down-regulation in the expression of *rhodopsin* in cavefish species (*52*).

However, how are the eyeless cave-dwelling *Leptonetela* spiders physiologically able to perceive light? On the basis of the results from the annotation of PPGs, nearly all genes encoding major components of the phototransduction pathway were present in both entrance and cave-dwelling species, i.e., opsins (*r-opsin*), which act as light receptors; G proteins such as Gα and Gβ, which are involved in the absorption of photons; PLC, which interacts with activated Gα; the trp protein, which plays an important role in PLC-mediated transient activation of the signalplex; and key genes such as *Arr* and *Gprk1*, which are involved in opsin deactivation and recycling (*53*–*57*). Unexpectedly, *rdgC* and *G*γ were absent in transcriptomes of all tested *Leptonetela* species. We cannot rule out the possibility that the absence of the two genes resulted from lineage-specific gene loss or difficult-to-detect levels of gene expression (*58*). Generally speaking, our results offer compelling evidence that cave-dwelling *Leptonetela* spiders displayed a nearly intact phototransduction pathway that was very likely derived from the original ocular phototransduction systems of ancestors, and this allows them to sense light, even without a functional eye structure. Since the external eyes have been documented to serve visual neuropils in the brain in spiders (*59*, *60*), further studies of the neuroanatomy of the brain are needed to confirm whether peripheral photoreceptors are completely absent in cave-dwelling spiders. Similarly, major components of the phototransduction pathway were also identified in the transcriptomes of cave-dwelling crustaceans (*61*). However, we were unable to determine whether extraocular photoreception existed in eyeless or eye-degraded cave-dwelling *Leptonetela* spiders, and thus further studies are required in the future.

Entrance spiders live in twilight zones, but cave-dwelling spiders live in deep caves with the complete absence of light. Thus, one may expect PPGs, and the phototransduction functions to be much more important for entrance spiders than cave-dwelling spiders. However, the results of our evolutionary analysis showed that all tested PPGs were under strong purifying selection in cave-dwelling spiders, and they were generally under similar levels of purifying selection in entrance and cave-dwelling spiders (Table 3). Similarly, a RELAX analysis did not detect significant signals of relaxed selection in most of the six tested PPGs of cave-dwelling species compared with their entrance relatives. However, *Arr*, which acts as a mediator of r-opsin inactivation and plays an essential role in the termination of the phototransduction cascade in vivo (*62*), was estimated to be under relaxed selective pressure in cave-dwelling spiders relative to entrance spiders. Intuitively, one may assume that the phototransduction system of cave-dwelling spiders has undergone relaxed selection. Nevertheless, the identified purifying selection on all tested PPGs of cave-dwelling spiders and entrance spiders strongly supported the hypothesis that their phototransduction systems were still under strong functional constraints. Therefore, we hypothesized that this may be due to different purposes of the interaction with light between the entrance and cave-dwelling spiders. Similar to other subterranean animals, cave-dwelling *Leptonetela* spiders spend their entire life cycles in a completely lightless environment (*63*, *64*), and, thus, it is reasonable that their phototransduction functions primarily contribute to discriminating the boundary between light and dark environments, thereby helping them to choose suitable habitats (*10*). However, entrance spiders live in environments with light-dark cycles. They should thus demonstrate more complex interactions with light due to activities such as foraging (*65*), mating (*66*), intraspecific communication (*67*), and development (*68*). Alternatively, the estimated relaxed selection on *Arr* may have resulted from algorithmic bias in RELAX. It is difficult to characterize changes in selective strength within short timescales using RELAX such as switches between purifying and positive selection (*35*).

Pleiotropy, occurring when a mutation or gene affects more than one phenotypic character (*69*), may result in strong purifying selection on genes that have lost their canonical function (*70*). However, Wang *et al.* (*71*) presented evidence that there was a low level of pleiotropy on a genome-wide scale in yeast, nematode, and mouse mutants. Thus, it is extremely unlikely that purifying selection on all PPGs has resulted from other biological functions than phototransduction. As a result, the phototransduction functions in cave-dwelling species appear to be under strong functional constraints, indicating that their maintenance has been shaped by purifying selection in cave-dwelling species. Consequently, our molecular evolutionary analysis suggested that all tested PPGs of cave-dwelling *Leptonetela* spiders are under strong purifying selection. This is compelling evidence supporting the natural selection hypothesis and rejecting the neutral evolution hypothesis.

In conclusion, we showed that cave-dwelling eyeless spiders still need to detect light, probably due to natural selection for avoiding the dry habitats outside of caves, and that they probably retained the original ocular phototransduction system derived from eyed ancestors. We hypothesize that retaining phototransduction functions may be more common in terrestrial subterranean animals than previously thought. This integrative study comprises an ideal system for studying the evolution of eyes and visual systems in subterranean animals. Our study also reveals a case of mismatch between phenotype and genotype or physiological function in a long-term evolutionary process, highlighting the risk of using phenotype to infer genotype or physiological function.

## MATERIALS AND METHODS

### Biological materials and collection

A total of 10 *Leptonetela* species were collected from karst caves or cave entrances on the Yunnan-Guizhou Plateau, China (table S1) (*72*–*75*). Five entrance species with six intact eyes including completely eye pigment (*L. curvispinosa*, *L. mengzongensis*, *L. palmata*, *L. pentakis*, and *L. reticulopecta*) (Fig. 1, B to F) were separately collected from the twilight zone of Yelaoda Cave, Mengzong Cave, Dixian Cave, Sanxing Cave, and Manwang Cave (table S1). Five cave-dwelling species, including two species with highly reduced eyes (*L. kangsa* and *L. sublunata*) that exhibited only six white spots without any eye pigment and three completely eyeless species (*L. danxia*, *L. hamata*, and *L. tetracantha*) (Fig. 1, G to K), were collected from the total darkness of Kangsa Cave, Manwang Cave, Shenxian Cave, Guanyin Cave, and Wengshuida Cave, respectively (table S1). All samples were collected from the spider webs and separately housed in 50-mm polystyrene petri dishes using moistened cotton to maintain humidity. Adult *Leptonetela* individuals for behavioral experiments were placed in their natural habitats. This research adhered to the ethical guidelines and legal requirements for animal treatment of Hubei University, and all animal experiments were approved by the Animal Care and Welfare Committee of Hubei University. We ensured gentle handling of the spiders throughout the experiments to minimize any negative impact on their welfare.

### Behavioral experiments of photoresponse

To determine whether cave-dwelling spiders and entrance spiders retained photoresponse behavior, we used light/dark choice tests (*13*) to investigate their photoresponse behavior using five entrance species (*L. curvispinosa*, *L. mengzongensis*, *L. palmata*, *L. pentakis*, and *L. reticulopecta*) (Fig. 1, B to F) and five cave-dwelling species (*L. danxia*, *L. hamata*, *L. kangsa*, *L. sublunata*, and *L. tetracantha*) (Fig. 1, G to K). A single tester was placed in a clean plastic box (30 cm in length, 20 cm in width, and 14.5 cm in height) that was divided into two fields, a bright area (B) and an equally large dark area (D), by black plastic. Spiders of each species were equally placed in the dark and light zones of boxes. We placed moistened cotton strips in each box to maintain humidity.

For both cave-dwelling and entrance species, we first performed the behavioral experiments in the twilight entrances of the inhabited cave for each species. A total of 20 individuals from each species were tested from 7:00 a.m. (0.5 hours before dawn) to 7:00 p.m. (0.5 hours before dusk), and then we recorded the final area for each individual in the plastic boxes. Afterward, to conduct control experiments, we repeated the tests after all individuals were transferred into totally dark zones of the same caves.

### Survival tests in the field and RH choice experiment in Y-tube

We then tested whether maintenance of photoresponse behavior could be the result of natural selection to avoid leaving the cave in eyeless or eye-degraded cave-dwelling *Leptonetela* spiders, and we performed survival tests in Manwang Cave where *Leptonetela* species live to examine whether the survival rates of cave-dwelling species were different from that of entrance species. We chose two entrance species (*L. palmata* and *L. reticulopecta*) (Fig. 1, D and F) living in the cave entrance and two cave-dwelling species (*L. danxia* and *L. sublunata*) (Fig. 1, G and J) living in dark zones without light to perform the experiments. Single individuals were placed in a plastic bowl (4 cm in diameter and 4.5 cm in height). For each species, adult individuals were collected and randomly divided into four treatments: (i) Individuals were placed in the twilight zone of the cave entrance without water supply; (ii) individuals were placed in the dark zone of the deep cave without water supply; (iii) individuals were placed in the twilight zone of the cave entrance with water supply; and (iv) individuals were placed in the dark zone of the deep cave with water supply. There were at least 10 individuals in each treatment for both species. We recorded the number of surviving individuals in each treatment after 24 hours.

To assess the presence of physiological humidity sensors in *Leptonetela* species, we conducted Y-tube humidity choice assays to investigate their capability to detect humidity levels using three entrance species (*L. curvispinosa*, *L. palmata*, and *L. pentakis*) (Fig. 1, B, D, and E) and three cave-dwelling species (*L. danxia*, *L. kangsa*, and *L. sublunata*) (Fig. 1, G, I, and J). A glass Y-tube (2.5 cm in diameter, 10-cm base arm, and two 10-cm arms at 70° to one another) setup was positioned horizontally in an illuminated room equipped with two 40-W light-emitting diodes. The room was maintained at a temperature of 25° ± 3°C and approximately 41.85 ± 2.25% RH. Airflow, purified through activated carbon, was introduced into two chambers (dry and wet chambers) using an air pump (SEBO AR-20, 2 × 3.5 liters/min). The airflow passed through two chambers (dry and wet chambers) and then through two flowmeters (SENLOD LZB-3W) with identical flow velocities (1.6 liters/min) before being directed into the two areas (dry and wet areas) of the Y-tube (Fig. 2A). In the wet chamber, RH was maintained at a level of 80.3 ± 4.2% using a saturated sponge. The dry chamber used a desiccated sponge to maintain an RH of 46.5 ± 5.7%. Meanwhile, the base arm was consistently maintained at an RH of 59.4 ± 6.5%. The RH levels were significantly different among two areas (dry and wet areas) and one base arm, respectively [analysis of variance (ANOVA) multiple comparison test: *P* < 0.001]. A minimum humidity difference of 20% was maintained between the wet and dry areas throughout the testing. Adult individual of *Leptonetela* spiders was transferred into Y-tube, and its locomotor activity was observed for a maximum of 5 min. Duration times were recorded when the tested individuals traveled at least 10 mm into the respective dry or wet area. If the tested individual did not move through the base arm within 5 min, then a duration time of 0 s was recorded. Using two stopwatches, we timed the duration times of each choice test and gathered data from a minimum of 20 individuals for each species. In addition, RH measurements were obtained from each area and base arm using a portable hygrothermograph (ASAIR) after evaluating five individuals from each *Leptonetela* species. After each test, water was added to the wet chamber to saturate the wet sponge. To prevent potential contamination from individual volatiles and chemicals, the Y-tube was cleansed with ethanol before every experiment. Furthermore, the left-right orientation of the Y-tube, air pump, and flowmeters was randomly switched to prevent bias (*76*, *77*).

### Transcriptome analysis

#### Taxon sampling and RNA extraction

Adult female spiders of five entrance species (*L. curvispinosa*, *L. mengzongensis*, *L. palmata*, *L. pentakis*, and *L. reticulopecta*) (Fig. 1, B to F) and five cave-dwelling species (*L. danxia*, *L. hamata*, *L. kangsa*, *L. sublunata*, and *L. tetracantha*) (Fig. 1, G to K) were randomly sampled from nine natural caves in Guizhou Province, China (table S1) and then placed in the lab of the School of Life Sciences at Hubei University at 16 to 18°C. The cave-dwelling and entrance species were starved separately under 12-hour dark:12-hour dark and 12-hour light:12-hour dark for at least 7 days to eliminate the influence of internal solute. Initially, we collected cephalothorax samples from six species (entrance species: *L. mengzongensis*, *L. palmata*, and *L. reticulopecta*; cave-dwelling species: *L. danxia*, *L. sublunata*, and *L. tetracantha*). To increase the sample size for analyses, we added whole-body samples from four more species (entrance species: *L. curvispinosa* and *L. pentakis*; cave-dwelling species: *L. hamata* and *L. kangsa*). All samples were promptly frozen in liquid nitrogen after collection. Subsequently, these samples were immediately stored at −80°C.

#### Transcriptome sequencing and de novo assembly

Total RNA was extracted from the cephalothorax of each *Leptonetela* individual using the TRIzol method (TIANGEN BIOTECH, Beijing) following the manufacturer’s protocol. For each sample, a paired-end library with an insert size of approximately 200 to 250 bp was constructed using the Illumina TruSeq RNA sample prep kit according to the manufacturer’s protocol (*78*). All libraries were sequenced commercially to generate paired-end reads of an average length of 150 bp on the Illumina NovaSeq 6000 sequencing platform (Beijing Allwegene Technology Company Limited, China). In addition, *C. californica* (Araneae: Leptonetidae) was selected as the outgroup species, and its transcriptome data were downloaded from National Center for Biotechnology Information (NCBI). We assessed the sequence quality of 11 transcriptomes from 10 *Leptonetela* species and one outgroup species (*C. californica*) using FastQC (v.10.1) (*79*) and trimmed out low-quality sequences, ambiguous sequences, or ploy-N and artificial sequences from raw reads using Trimmomatic (v.0.33) (*80*). A total of 11 de novo transcriptome assemblies were conducted by Trinity (v.2.8.5) with default parameters (*81*). We used BUSCO (v.5.3.2) to assess the completeness of the transcriptome assemblies and used OrthoDB’s Arthropoda database of orthologous groups (*n* = 1013) as a reference dataset (*82*). The longest contig of each gene was selected to be a unigene for downstream analyses, and redundant contigs were removed (*52*).

### Ortholog identification and phylogenetic reconstruction

The open reading frames (ORFs) of each transcript were predicted using TransDecoder (v.5.5.0). We inferred single-copy orthologous genes among the 11 transcriptomes by OrthoFinder (v.2.5.4) (*83*, *84*). All putative orthologous sequences were aligned by PRANK (v.170427) (*85*), and poorly aligned sites and divergent regions were filtered by GBLOCKS (v.0.91b) (*86*). In addition, we discarded alignment sequences with aligned regions shorter than 100 nucleotides (*87*). All single-copy orthologous genes were concatenated to a super alignment matrix and then were used to reconstruct a phylogenetic tree under the maximum-likelihood (ML) framework using IQ-TREE software (v.2.1.2) (*88*) with 1000 bootstrap replicates by the best-fitting nucleotide substitution model “GTR + GAMMA”, which was predicted using the jModelTest program (v.2.1.4) according to Bayesian information criteria (*89*). Because of the large scale of our transcriptomic dataset, certain computationally intensive dating methods, such as those integrated in BEAST, were deemed impractical. Therefore, we used MCMCTREE in PAML 4.7 instead to estimate the divergence times between species based on the super alignment matrix aforementioned with the “independent rates” option and the best-fitting substitution model “GTR + G” that was determined by jModelTest. A Markov chain Monte Carlo analysis was running for 500,000 generations, using a burn-in of 50,000 iterations. Because of the unavailability of fossil records for these species, we resorted to using secondary calibrations informed by the results of a previous study that are 110.86 Ma ago with a 95% HPD between 80.24 and 144.66 Ma ago for *Calileptoneta* group and 19.68 Ma ago with a 95% HPD between 11.39 and 29.00 Ma ago for *Leptonetela* group (*31*).

### Annotation of PPGs

We used a suite of phylogenetic tools to identify the PPGs in the ORFs of 10 de novo assembly transcriptomes of *Leptonetela* species. Putative PPG sequences were identified from the ORFs by comparing with a well-characterized PPG dataset from the Light Interaction Toolkit (LIT) (v.1.1) of PIA tool using TBLASTN with an *E* value cutoff of 1 × 10^−5^ (*34*). Then, we used the resulting coding sequence (CDS) to search against the nonredundant protein database blasted on BLASTX (*90*). Only CDSs with the best hits of PPGs were retained for subsequent analysis. Last, sequences and alignment of each retrieved gene were checked manually. We retained 11 PPGs annotated in at least one *Leptonetela* species, and the fragment sequences within the same ORF of the same species were connected by filling the missing parts with “N.”

### Annotation of spider opsins and quantitative reverse transcription polymerase chain reaction

We further identified opsins, which were initiators and essential components of the phototransduction cascade in the ORFs of 10 de novo assembly transcriptomes of *Leptonetela* species using sequence similarity and phylogenetic approaches. Using a previously established compilation of conserved spider opsins as the query sequence source, the accession numbers of opsins in NCBI databases were as follows: jumping spider (*Hasarius adansoni*) (Rh1, 6I9K_A; Rh2, BAG14331.1; Rh3, BAG14332.1; Rh4, BAO73879.1; peropsin, BAJ22674.1); jumping spider (*Plexippus paykulli*) (Rh1, BAG14333.1; Rh2, BAG14334.1; Rh3, BAG14335.1); wandering spider (*Cupiennius salei*) (Rh1, CCO61973.1; Rh2, CCO61974.1; Rh3, CCO61975.1; arthropsin, CCP46951.1; c-opsin, CCP46950.1; peropsin, CCP46949.1); orb spider (*Araneus ventricosus*) (Rh7, GBM12885.1); pea aphid (*Acyrthosiphon pisum*) (c-opsin, AWM72030.1); and red flour beetle (*Tribolium castaneum*) (c-opsin, NP_001138950.1) (*24*). We searched the opsins from transcriptome assemblies of the 10 *Leptonetela* species using TBLASTN with an *E* value cutoff of 1 × 10^−5^ (*34*). Then, we used the resulting CDS to search against the nonredundant protein database blasted on BLASTX (*90*). Last, we reconstructed a phylogenetic tree of opsins using IQ-TREE (*88*) with 1000 bootstrap replicates by the best-fitting model “LG + F + R4,” which was predicted using the ProtTest3 (v.3.4.2) (*91*) according to Bayesian information criteria and three c-opsin sequences as the outgroup.

We sampled populations of the 10 *Leptonetela* species from their native caves and placed in individual tubes and subsequently transferred into liquid nitrogen. The total RNA was extracted from multiple individuals of each species and was treated using TRIzol reagent (TIANGEN BIOTECH, Beijing), following the manufacturer’s protocol. We performed three biological replicates with five adults per replicate for each species. Thus, a total of three independent RNA extractions were conducted for each species for quantitative reverse transcription polymerase chain reaction (qRT-PCR) of *Rh2*. Two micrograms of RNA was reverse-transcribed to obtain cDNA using EasyScript One-Step gDNA Removal and cDNA Synthesis SuperMix (TIANGEN BIOTECH, Beijing), following the manufacturer’s instructions. We analyzed each species in triplicate by qRT-PCR using SYBR Green Master Mix (YEASEN, China). We designed the specific primer of *Rh2* based on the locations with common sequences in the 10 *Leptonetela* species derived from our de novo assembled transcriptome (table S6). To check reproducibility, we performed qRT-PCR for each sample with three technical replicates. We chose the β*-actin* gene as a reference gene. We compared the relative expression levels directly using the 2^−*ΔΔC*_T_^ method and used β*-actin* for normalization (*92*, *93*).

### Evolutionary analysis on PPGs of *Leptonetela* spiders

To verify that PPGs are under purifying selection and thus are putatively functional in cave-dwelling spiders and to test the possibility of differential selection on PPGs between entrance spiders and cave-dwelling spiders, we estimated the ω values [nonsynonymous/synonymous rate ratio (*d*_N_/*d*_S_)] using a likelihood approach. We selected six PPGs that were annotated in a minimum of three entrance and three cave-dwelling *Leptonetela* species, respectively, for molecular evolutionary analysis. To each selected PPG, the sequences retained were required to have a length of over 100 amino acids and cover more than 50% of the full ORF. The identified nucleotide acid sequences of PPGs were aligned on the basis of codons using CLUSTALX (v.2.1) (*94*, *95*). The phylogenetic tree of *Leptonetela* species was taken from our previously generated ML tree. Nonsynonymous (*d*_N_) and synonymous (*d*_S_) nucleotide substitution rates (ω) were estimated using the likelihood method implemented in the CODEML program in PAML (v.4.1) (*96*). Likelihood ratio tests were conducted in the R program (v.4.0.5) (*97*). In addition, we used the program RELAX (v. 2.5.48) to examine whether the cave-dwelling spiders have undergone relaxed selection pressure (*35*).
