# Supplementary material for: Eyeless cave-dwelling Leptonetela spiders still rely on light
Source: Sci Adv. 2023 Dec 20;9(51):eadj0348. doi: 10.1126/sciadv.adj0348 (PMC10732526; doi:10.1126/sciadv.adj0348)
Supplement: Supplementary file 1 — Tables S1 to S6 Legends for movies S1 and S2 [file sciadv.adj0348_sm.pdf]

Science Advances
AAAS

Supplementary Materials for

Eyeless cave-dwelling *Leptonetela* spiders still rely on light

Kai Wang *et al.*

Corresponding author: Jie Liu, jieliu@hubu.edu.cn

Sci. Adv. **9**, eadj0348 (2023)
DOI: 10.1126/sciadv.adj0348

The PDF file includes:

Tables S1 to S6
Legends for movies S1 and S2

Other Supplementary Material for this manuscript includes the following:

Movies S1 and S2

Table S1. Ecological conditions of collection sites.

Species	Collection Sites	Locations	Ecologies	Habitats	Eye Morphotypes	Categories
L. curvispinosa	Twilight entrance of Yelaoda Cave, Baiguo Village, Wenge Town, Dafang County, Bijie City, Guizhou Province	27.10°N, 105.28°E	Epigean	Cave entrance	Six intact eyes	Macrophthalmic species
L. danxia	Dark zone of Shenxian Cave, Shiliping Village, Zhudong Town, Pan County, Liupanshui City, Guizhou Province	28.62°N, 104.76°E	Troglobiont	Deep cave	Eyeless	Anophthalmic species
L. mengzongensis	Twilight entrance of Mengzong Cave, Liangfengya Village, Chumi Town, Tongzi County, Zunyi City, Guizhou Province	28.13°N, 106.82°E	Epigean	Cave entrance	Six intact eyes	Macrophthalmic species
L. hamata	Dark zone of Guanyin Cave, Jiangjun Bay, Hongfenghu Town, Qingzhen City, Guiyang City, Guizhou Province	26.28°N, 106.25°E	Troglobiont	Deep cave	Eyeless	Anophthalmic species
L. palmata	Twilight entrance of Dixian Cave, Guanping Village, Donghuang Town, Xishui County, Zunyi City, Guizhou Province	28.47°N, 106.53°E	Epigean	Cave entrance	Six intact eyes	Macrophthalmic species
L. kangsa	Dark zone of Kangsa Cave, Geyi Town, Taijiang County, Kaili City, Guizhou Province	26.79ºN, 108.21ºE	Troglobiont	Deep cave	Highly reduced	Microphthalmic species
L. pentakis	Twilight entrance of Sanxing Cave, Hongguang Village, Yankong Town, Jinsha County, Bijie City, Guizhou Province	27.30°N, 106.17°E	Epigean	Cave entrance	Six intact eyes	Macrophthalmic species
L. sublunata	Dark zone of Manwang Cave, Dishui Village, Kuankuo Town, Suiyang County, Zunyi City, Guizhou Province	28.24°N, 107.28°E	Troglobiont	Deep cave	Highly reduced	Microphthalmic species
L. reticulopecta	Twilight entrance of Manwang Cave, Dishui Village, Kuankuo Town, Suiyang County, Zunyi City, Guizhou Province	28.24°N, 107.28°E	Epigean	Cave entrance	Six intact eyes	Macrophthalmic species
L. tetracantha	Dark zone of Wengshuida Cave, Tiesuoyan Village, Guiding County, Qiannan Buyi and Miao Autonomous Prefecture, Guizhou Province	26.36°N, 107.17°E	Troglobiont	Deep cave	Eyeless	Anophthalmic species

Table S2. Statistics of assembled contigs for 10 individuals of *Leptonetela* species in this study. Abbreviation: nt, nucleotide (s).

Species	Number of bases (nt)	Number of reads	N50 statistics (nt)	Mean length (nt)	GC count
L. curvispinosa	107,256,066	123,738	1,612	741	38%
L. danxia	157,378,188	162,064	2,049	971	37%
L. mengzongensis	97,632,539	136,140	1,227	717	37%
L. hamata	137,619,331	148,647	1,825	825	38%
L. palmata	96,996,826	162,737	921	596	38%
L. kangsa	149,145,732	163,720	1,819	799	38%
L. pentakis	113,842,377	126,684	1,732	739	38%
L. sublunata	116,092,958	142,735	1,628	813	37%
L. reticulopecta	101,678,089	144,853	1,210	702	37%
L. tetracantha	93,950,515	147,578	1,034	637	37%

Table S3. BUSCO assessment for the transcriptomes for ten individuals of *Leptonetela* species.

Species	Complete BUSCOs (%)	Fragmented BUSCOs (%)	Missing BUSCOs (%)	Total BUSCOs searched
L. curvispinosa	92.2	4.2	3.6	1013
L. danxia	93.3	3.1	3.6	1013
L. mengzongensis	82.1	6.8	11.1	1013
L. hamata	93.0	3.4	3.6	1013
L. palmata	76.3	10.3	13.4	1013
L. kangsa	90.7	3.4	5.9	1013
L. pentakis	92.5	4.2	3.3	1013
L. sublunata	94.8	2.4	2.8	1013
L. reticulopecta	93.9	3.1	3.0	1013
L. tetracantha	79.5	8.5	12.0	1013

Table S4. The GenBank accession numbers on PPGs of *Leptonetela* species.

Species	*Arr*	*DAGK*	*Gprk1*	*Gprk2*	*rdgC*	*PKC*	*PLC*	*rdgB*	*Gγ*	*trp*	*Gα*	*Gβ*	*r-opsin*
L. curvispinosa	OR735630	OR735640	OR735647	OR735656	NA	NA	OR735677	OR735687	NA	NA	OR735702<br>OR735711	OR735719<br>OR735737<br>OR735730	OR735661
L. danxia	OR735631	OR735641	OR735648	OR735657	NA	OR735671	OR735678	OR735688	NA	NA	OR735703<br>OR735712	OR735726<br>OR735735	OR735662
L. mengzongensis	OR735634	OR735643	OR735651	OR735659	NA	NA	OR735681	OR735691	NA	OR735698	OR735705	OR735727	OR735665
L. hamata	OR735632	OR735642	OR735649	OR735658	NA	OR735672	OR735679	OR735689	NA	OR735696	OR735713	OR735723	OR735663
L. palmata	OR735635	OR735644	OR735652	NA	NA	NA	OR735682	OR735692	NA	OR735699	OR735706	OR735722<br>OR735731	OR735666
L. kangsa	OR735633	NA	OR735650	NA	NA	OR735673	OR735680	OR735690	NA	OR735697	OR735704<br>OR735714	OR735725<br>OR735732	OR735664
L. pentakis	OR735636	NA	OR735653	OR735660	NA	NA	OR735683	OR735693	NA	NA	OR735715<br>OR735707	OR735720<br>OR735734<br>OR735729	OR735667
L. sublunata	OR735638	OR735645	OR735655	NA	NA	OR735675	OR735685	OR735694	NA	OR735701	OR735717<br>OR735710	OR735721<br>OR735728<br>OR735733	OR735669
L. reticulopecta	OR735637	NA	OR735654	NA	NA	OR735674	OR735684	NA	NA	OR735700	OR735708<br>OR735716	OR735724<br>OR735736	OR735668
L. tetracantha	OR735639	OR735646	NA	NA	NA	OR735676	OR735686	OR735695	NA	NA	OR735709<br>OR735718	OR735738	OR735670

NA indicates that the gene was not identified in the transcriptome of *Leptonetela* species.

Table S5. Selection tests conducted by RELAX on PPGs of *Leptonetela* species.

RELAX	Test branches	Reference branches	Models	Ln *L*	2Δ (Ln *L*)	K *	#. Params	*P* value
r-opsin	Cave-entrance lineages	Deep-cave lineages	Null	-2826.9			36	
			Alternative	-2826.4	1.04	0.90	37	0.308
			Partitioned Exploratory	-2825.6	2.60		41	0.761
rdgB	Cave-entrance lineages	Deep-cave lineages	Null	-8070.8			30	
			Alternative	-8070.0	1.44	0.46	31	0.230
			Partitioned Exploratory	-8069.5	2.42		35	0.788
PLC	Cave-entrance lineages	Deep-cave lineages	Null	-6930.0			36	
			Alternative	-6928.5	2.96	0.34	37	0.085
			Partitioned Exploratory	-6928.3	3.36		41	0.645
Gβ	Cave-entrance lineages	Deep-cave lineages	Null	-2311.6			31	
			Alternative	-2310.8	1.54	1.30	32	0.215
			Partitioned Exploratory	-2310.7	1.74		36	0.884
Gα	Cave-entrance lineages	Deep-cave lineages	Null	-1819.7			33	
			Alternative	-1819.7	0.02	1.65	34	0.888
			Partitioned Exploratory	-1818.5	2.46		38	0.783
Arr	Cave-entrance lineages	Deep-cave lineages	Null	-2680.2			33	
			Alternative	-2678.2	4.02	0.58	34	**0.045**
			Partitioned Exploratory	-2677.9	4.72		38	0.451

*K is the selection intensity parameter. *P* values lower than 0.05 are shown in bold.

Table S6. Primer sequences for this study.

Gene Symbols	qRT-PCR primers
Rh2	Forward Primer: 5'-CCGAAGGCAACATGACTAGCTG-3'<br>Reverse Primer: 5'-TGGCGATCTTGGTGAGTCTGA-3'
β-actin	Forward Primer: 5'-CCCTGAGAAAGTACTCCGT-3'<br>Reverse Primer: 5'-ATCCACATCTGCTGGAAGGTG-3'

Movie S1. Cave-dwelling spider (*L. kangsa*) is drinking water in the lab.

Movie S2. Relative humidity choice experiments for *Leptonetela* species in the lab.
